# Cellular Function of TRIM E3 Ubiquitin Ligases in Health and Disease

**DOI:** 10.3390/cells11020250

**Published:** 2022-01-12

**Authors:** Germana Meroni, Solange Desagher

**Affiliations:** 1 Department of Life Sciences, University of Trieste, 34127 Trieste, Italy; 2 Institut de Génétique Moléculaire de Montpellier, University Montpellier, CNRS, Montpellier, France

The field of the Tripartite Motif (TRIM) family has progressively attracted increasing interest during the last two decades. The presence of a distinctive amino-terminal module, composed of a RING, one or two B-box(es) and Coiled-coil domains, is the distinctive hallmark of the TRIM family members [1]. Several reports demonstrated that the shared TRIM proteins’ domain composition is associated with their involvement in the ubiquitination process and, indeed, TRIM proteins act as E3 ubiquitin ligases for the specific recognition of substrates to be modified. Notably, TRIM family members are implicated in many clinically relevant physiological processes and in various pathological conditions. Today, many efforts are directed towards the prospect of recognizing TRIM proteins/functions as therapeutic targets. 

This Special Issue of *Cells* is dedicated to the basic involvement of specific TRIM family members in different pathologies, the development of therapeutic approaches, to finally outline the possible commercial exploitation of TRIM-related findings.

TRIM17 function is thoroughly overviewed in this Special Issue. TRIM17 acts in connection with other family members within a TRIM-network including TRIM39, 41, 44, and 28. The cross-regulation between TRIM17 and other TRIM members implicates the formation of inactive TRIM hetero-complexes that inhibits their respective E3 ubiquitin ligase activity. This contributes to the finely tuned control of mitosis, of ZSCAN21- and NFATc3-dependent transcription, of selective autophagy and apoptosis [2]. Besides the involvement in several cellular processes, TRIM17 is potentially implicated in the pathogenesis of Parkinson’s disease (PD). Indeed, by controlling ZSCAN21 activity, TRIM17 regulates the expression of the pre-synaptic protein α-synuclein, the most important player in PD. This is supported by the identification of PD-associated genetic variants in the *TRIM17* gene. Furthermore, genomic studies implicate *TRIM17* de novo mutations in autism spectrum disorders. Given TRIM17’s involvement in proliferation and apoptosis, it is not surprising that several findings also implicate it in tumorigenesis and chemoresistance in a context-specific manner, as often occurs in cancer. 

In support of the pleiotropic function of TRIM E3 ubiquitin ligases, another example is given by TRIM8 reviewed in this issue [3]. TRIM8 has been shown to act as a tumor suppressor in several types of cancers, from glioblastoma to breast cancer. This role is likely exerted through the stabilization and activation of p53 and the parallel degradation of ∆Np63α, a pro-proliferative paralogue of p53. However, TRIM8 can also act as an oncogene promoting proliferation and invasiveness through the activation of the NFκB pathway by promoting PIAS3 translocation and Lys63-linked polyubiquitination of TAK1. Furthermore, TRIM8 induces the activation of the JAK-STAT pathway through the degradation of two STAT protein inhibitors, PIAS3 and SOCS-1. Besides cancer involvement, the critical role of TRIM8 in several immunological-related pathological processes is outlined. As a third area of TRIM8 clinical involvement, mutations in its gene have been detected in diverse genetic disorders: infantile epileptic encephalopathy and Focal Segmental Glomerulo Sclerosis syndrome [3].

Several other TRIM family members are found to be implicated in tumors. In particular, many TRIM proteins play a relevant role in hormonal cancer types, as thoroughly reviewed in this Special Issue [4]. TRIM proteins play a role in breast cancer, prostate cancer, ovarian and endometrial cancers via their action in different molecular pathways. Very often, the same TRIM members can function via different mechanisms. A pivotal role in hormone-dependent cancers is elicited by the PHD-BROMO domain sub-group of TRIM proteins (TRIM24, 28, 33) and by TRIM25 but other family members are also implicated. The mechanisms affected by TRIM proteins are manifold: direct interaction with the hormone receptors, ER and AR; regulation by ubiquitination, as expected for E3 ubiquitin ligases; epigenetic control; Epithelial to Mesenchymal Transition; cell cycle and apoptosis regulation; DNA damage control; and involvement in the main cancer-related pathways, e.g., p53, Jak/STAT to name a few. Common functions of TRIM proteins in hormone-driven cancers are highlighted. Indeed, improved understanding of the biological impact of TRIM proteins may pave the way for improved prognosis and novel therapeutics [4].

Innate immunity is a major role of many members, if not all, of the Tripartite Motif family. Indeed, TRIM proteins are able to elicit pathogen-associated molecular patterns (PAMP) response upon viral and bacterial infections. In this issue, the antiviral role of TRIM22 is reviewed, as an example of several TRIM E3 ligases with similar functions [5]. In particular, it is outlined that TRIM22 restricts HIV-1 by favoring the maintenance of a state of proviral latency and by repressing its transcription, whereas TRIM22 activity in the ubiquitination pathway is necessary to interfere with the release of particles. In addition, TRIM22 acts as a restriction factor of many other viruses, both RNA and DNA viruses. This is discussed in light of the different mechanisms involved, from ubiquitination to induction of relevant viral cycle components via direct interactions to transcription repression and epigenetic control. The mechanism of TRIM22 antiviral activity varies depending on the viral target, and almost any domain of the protein can execute antiviral activity [5]. 

Rare genetic diseases, implicating neurological and neuromuscular systems, can be caused by loss-of-function mutations in TRIM32 and in the TRIM-like E3 ligase Malin [6]. TRIM32 is involved in a form of Limb Girdle Muscular Dystrophy characterized by progressive muscle weakness, whereas Malin is mutated in a form of progressive myoclonus epilepsy. The two proteins share a common C-terminal domain often altered by genetic mutations. They target several substrates for ubiquitination, thereby driving different fates and affecting several different pathways and mechanisms that are reviewed in light of the disease they are involved in. However, the composition of the domain shared by TRIM32 and Malin and their close evolutionary origin are in line with the observation that the two proteins are involved in common pathways such as autophagy, although with a context-dependent outcome, Wnt pathway and glucose metabolism [6]. 

Another TRIM E3 ubiquitin ligase with an important function in muscle mass homeostasis is MuRF1/TRIM63. It is involved in catabolic states characterized by dramatic skeletal muscle wasting due to the targeting for degradation of several myofibrillar proteins, including the main contractile proteins alpha-actin and myosin heavy chain (MHC) [7]. In this issue, data are reported in support of the role of UBE2L3, an E2 conjugating enzyme that cooperates with MuRF1/TRIM63 on alpha-actin and MHC degradation in catabolic C2C12 myotubes. In this work, using MicroScale-Thermophoresis, a detailed analysis on the strength of the interaction between MuRF1/TRIM63 and the contractile proteins important in skeletal muscle atrophy is reported, in the presence or absence of the E2 conjugating enzyme UBE2L3. The data show that both MuRF1 and E2L3 are important regulators of muscle mass. The two proteins potentially act via several mechanisms, although the exact role of each partner remains to be established, as the presence of the substrate adds complexity to the mechanisms involved. 

Mitsugumin 5 (MG53) is another TRIM family member, also known as TRIM72, that is expressed mainly, but not exclusively, in muscle tissues, primarily skeletal and cardiac cells. Within the cells and through its redox-dependent oligomerization, MG53/TRIM72 is able to repair membranes at injury sites by facilitating fusion of vesicles translocating from the cytoplasm to the damaged membrane. In this Special Issue, Li et al. provide an overview of the pathological implication of MG53/TRIM72 defective animal models that present with skeletal muscle and cardiomyocyte repair defects, and increased susceptibility to myocardial infarction. In addition, these mouse models display increased vulnerability to stress and injury in lung, kidney and cornea, tissues where MG53/TRIM72 is expressed. More importantly, the use of MG53/TRIM72 as therapeutic agent is highlighted. Indeed, transgenic mice expressing and secreting MG53/TRIM72 show enhanced abilities of tissue regeneration. Furthermore, muscle-specific overexpression of the human gene via adeno-associated virus (AAV), as well as systemic delivery of the recombinant human protein (rhMG53), are able to boost membrane repair in cardiomyocytes and protect cardiac cells from ischemia/reperfusion. Consistently, intravenous injection of rhMG53 to mice protects injured muscles and improves skeletal muscle dystrophy. In addition, MG53/TRIM72-mediated repair machinery protects against injury in non-muscle organs, e.g., lung, kidney, liver and cornea. In conclusion, muscle-derived MG53/TRIM72 can indeed function as a myokine to facilitate repair of injury not only in muscle cells but also in remote organs reached through blood circulation [8]. 

TRIM-edicine Inc. has been established to commercially exploit the use of MG53/TRIM72 as a therapeutic agent in regenerative medicine. It is part of the landscape of biotech startups originating from the field of E3 ubiquitin ligase biology [9]. This scenery has been thoroughly presented in this Special Issue where the birth and the evolution of Targeted Protein Degradation therapeutic approaches are critically outlined, e.g., PROTAC, from concept to clinical trials or ‘classical’ inhibitory compounds. Within this field, TRIM E3 ubiquitin ligases are excellent targets to be exploited therapeutically for their involvement, as briefly presented above, in several pathological conditions, from cancer to genetic diseases. According to Bhaduri et al., there is immense opportunity for developing new drugs targeting the substrate binding domains of TRIM proteins, exploiting the homo/heteromeric interactions among TRIM family members, and the possibility of interfering with the pathways of their E3 ligase activity. At the same time, the unavailability of the structures of most TRIM proteins and the need to further elucidate their target substrates have so far relented the path into the pharmaceutical industry [9]. We strongly believe that in the next future, scientific and entrepreneurial initiative will boost the development of drugs against TRIM proteins or their use as E3 ubiquitin ligases for targeted protein degradation. 
